# Nanocellulose-Based Film-Forming Hydrogels for Improved Outcomes in Atopic Skin

**DOI:** 10.3390/pharmaceutics15071918

**Published:** 2023-07-10

**Authors:** Katarina Bolko Seljak, Barbara Sterle Zorec, Mirjam Gosenca Matjaž

**Affiliations:** Faculty of Pharmacy, University of Ljubljana, Aškerčeva cesta 7, 1000 Ljubljana, Slovenia; katarina.bolko-seljak@ffa.uni-lj.si (K.B.S.); barbara.sterle.zorec@ffa.uni-lj.si (B.S.Z.)

**Keywords:** film-forming hydrogels, nanocrystalline cellulose, betamethasone dipropionate, SMEDDS, atopic dermatitis

## Abstract

Atopic dermatitis (AD) is a chronic inflammatory skin disease characterized by impaired skin barrier function. Amongst the various dermal formulations that are being used and/or investigated for AD treatment, one of the advanced approaches is the use of hydrogels as film-forming systems that are applied directly to the skin and have the added value of providing a physical barrier, which is lacking in atopic skin. Novel film-forming hydrogels based on two different nanocrystalline celluloses (NCCs) in combination with one of two natural polymers (alginate or pectin) were developed for incorporation of betamethasone dipropionate (BDP). Initially, the low water solubility of BDP was resolved by prior dissolution in a self-microemulsifying drug delivery system (SMEDDS). The mixture of Kolliphor^®^ EL/Capryol^®^ 90 in a ratio of 8/2 was chosen on the merit of its high BDP-saturated solubility and no BDP precipitation upon water dilution, enabling BDP to remain dissolved after incorporation into hydrogels. The solvent evaporation method was used to prepare the films, and their high water retention capacity was confirmed in vitro on artificial membranes and pig ear skin. The presented results thus confirm NCC-based film-forming hydrogels as a very promising drug delivery system for AD treatment.

## 1. Introduction

Atopic dermatitis (AD), also known as atopic eczema, is a chronic relapsing inflammatory skin disease with a lifetime prevalence of up to 30% in children and 10% in adults and has been increasing over the past few decades worldwide [1,2].

Although the pathological process of this disease is not fully understood, it is the result of a complex interaction between skin barrier dysfunction and immune system dysregulation involving genetic, environmental, and infectious factors [2,3,4]. AD, as a very heterogeneous disease, is manifested in a wide clinical spectrum that includes different lesion morphologies and a distribution pattern that varies according to the age of the patient. In addition to skin lesions and pruritus, which are usually the most distressing symptoms, patients with AD often suffer from xerosis, skin pain, and sleep disturbances that severely impair their quality of life. The disease is also strongly associated with other atopic comorbidities, such as asthma and allergic rhinitis, and with non-atopic comorbidities, namely, psychological disorders such as anxiety and depression [1,3].

Approaches to AD treatment focus on restoring the skin barrier, controlling itching, reducing inflammation, and preventing or reducing potential infections [2]. In terms of AD relapse prevention, it is important to take non-pharmacological measures, such as daily skin care, regular bathing, and avoiding contact with causative allergens [2,4].

Despite newer nonsteroidal topical therapies, such as calcineurin inhibitors [5], topical corticosteroids remain the gold standard for the treatment of active eczematous diseases and the management of exacerbations due to their proven efficacy and safety when used appropriately [1]. In addition, the xerotic, sensitive skin of AD patients requires the use of nonirritating and nondrying topical vehicles [6]. In this sense, in addition to steroid treatment, the guidelines for the treatment of AD recommend skin hydration therapy with moisturizers to alleviate the frequent side effects and xerosis [7,8].

To date, various dermal formulations have been investigated for the treatment of AD [9]. One of the innovative approaches is the use of hydrogels as film-forming systems in dermal application [10]. Namely, the hydrogel is applied directly to the skin, forming a thin, transparent film after the evaporation of the solvent in situ [10]. Although they are primarily proposed for prolonged administration, the added value of film-forming hydrogels is their ability to provide a physical barrier on the skin surface that is lacking in atopic skin. Among other advantages, hydrogels contain a high percentage of aqueous medium, making them a superior vehicle that could alleviate skin dryness and further improve therapeutic efficacy [11].

Various biocompatible and biodegradable polymers can be used for hydrogel preparation [12], one of them being nanocrystalline cellulose (NCC), which is gaining interest due to its excellent and attractive mechanical and chemical properties as well as its renewability [13,14]. However, its properties may differ based on the source and manufacturing process [15].

In the present study, novel film-forming hydrogels were developed based on two different NCCs in combination with one of the natural polymers, using betamethasone dipropionate (BDP) as a glucocorticoid derivative, which is widely used in the treatment of AD. BDP has a 30-fold stronger anti-inflammatory effect than hydrocortisone, including having a prolonged duration of action [16,17] but low water solubility. Since hydrophobic compounds in hydrogel matrices have limited load-bearing capacity, the incorporation of poorly water-soluble drugs should be considered [18,19]. One way to accomplish this is to introduce molecules that can form inclusion complexes or to incorporate hydrophobic components into the hydrogel structure. Other possibilities include using hydrogels that contain micelles or nanoparticles in their structures [20] or combining the particles with a hydrogel and incorporating liposomes, nanoparticles, or microspheres [5,21]. Another way to prepare a hydrogel carrier for hydrophobic drugs is to compose a mixed micellar gel consisting of a polymer and a surfactant [22]. Moreover, another option is to dissolve the drug in a microemulsion [23,24] which can later be incorporated into the hydrogel.

Within our study, the low water solubility of BDP was solved by previously dissolving the drug in a self-microemulsifying drug delivery system (SMEDDS) and further incorporating it into the hydrogel formulation. The hydrogels thus prepared were later evaluated by differential scanning calorimetry for solubility assessment, while rheological measurements investigated the influence of the NCC type and of having an SMEDDS on the rheological features of hydrogels. The solvent evaporation method was used to convert hydrogels into films, and, as the pivotal goal of the present study, they were evaluated in vitro for their capacity for water retention on artificial membranes as well as their ability to decrease transepidermal water loss (TEWL) on pig ear skin. The ability of the formulation to form an artificial barrier on atopic skin that effectively reduces TEWL would distinguish NCC-based film-forming hydrogels as the superior drug delivery system in treatment of AD. In addition, BDP in vitro permeation from hydrogels as well as a cytotoxicity evaluation of NCCs as novel excipients were performed to thoroughly address the potential of NCC-based film-forming hydrogels as delivery systems for AD.

## 2. Materials and Methods

### 2.1. Materials

Gel NCC ((gNCC) Navitas, Podcerkev, Slovenia), powder NCC ((pNCC) Celluforce, Montreal, QC, Canada), sodium alginate ((ALG) Protanal^®^ LF 10/60, FMC BioPolymer, Philadelphia, PA, USA), pectin ((PEC) Sigma-Aldrich, St. Louis, MO, USA), glycerol (Pharmachem, Ljubljana, Slovenia), and purified water were used to prepare the film-forming hydrogels. Capryol^®^ 90 (Gattefosse, Saint-Priest, France) and Kolliphor^®^ EL (BASF, Ludwigshafen, Germany) were used to form a self-microemulsifying drug delivery system (SMEDDS). For the solubility study of betamethasone dipropionate ((BDP) Sicor S.R.L., Roveretto, Italy), oleic acid (Sigma-Aldrich, St. Louis, MO, USA), Plurol^®^ Oleique (Gattefosse, Saint-Priest, France), isopropyl myristate (Sigma-Aldrich, USA), Tween 80 (Merck, Darmstadt, Germany), Tween 20 (Merck, Darmstadt, Germany), Labrasol (Gattefosse, Saint-Priest, France), and polyethylene glycol 400 (Sigma-Aldrich, St. Louis, MO, USA) were used as excipients. For HPLC (high-performance liquid chromatography) analysis, all reagents (acetic acid (Merck, Darmstadt, Germany), methanol (Avantor, Radnor, PA, USA), and acetonitrile (Avantor, Radnor, PA, USA)) were of HPLC grade.

### 2.2. Preparation of SMEDDS

#### 2.2.1. Solubility Study

The solubility of BDP in various oil excipients (oleic acid, Capryol 90, Plurol^®^ Oleique, isopropyl myristate), surfactants (Kolliphor EL, Tween 80, Tween 20, Labrasol, polyethylene glycol 400), and water mixtures was determined by adding an excess amount of the drug to a few ml of selected excipients. The solutions were first stirred with a glass rod to moisture the drug, and then they were further stirred at room temperature for 48 h to reach equilibrium. The equilibrated samples were removed from the shaker and centrifuged at 3500 rpm for 15 min (for water mixtures) or at 50,000 rpm for 40 min (for oil and the surfactant excipient) at 25 °C. The supernatant was removed and filtered through a 0.45 μm membrane filter (Sartorius, Göttingen, Germany). The concentration of BDP was determined by the HPLC method, with samples tested in duplicate.

#### 2.2.2. HPLC Analysis

The amount of BDP was analyzed with the HPLC method using Agilent 1100 Series HPLC system (Agilent Technologies, Santa Clara, CA, USA). Samples were prepared differently based on their properties. Namely, for the water samples, 1 mL of the filtered supernatant (see Section 2.2.1) was transferred to a 10 mL volumetric flask which was then filled up with a 0.1% volume/volume (*v*/*v*) acetic acid solution in methanol. For the oil and surfactant samples, 100 mg of the supernatant was weighed accurately into a 50 mL volumetric flask and diluted accordingly with a 0.1% (*v*/*v*) acetic acid solution in methanol. A total of 10 μL of prepared samples were then analyzed using column XTerra^®^ RP18 (5μm 4.6 μm × 250 mm, Waters, Milford, MA, USA). The mobile phase consisted of a mixture of acetonitrile:bidistilled water (3:2 (*v*/*v*)) with 1.2 mL/min flow rate. The detector wavelength was set at 254 nm. All operations were carried out at 25 °C.

#### 2.2.3. Construction of Phase Diagram

Based on solubility studies, a pseudo-three-component phase diagram was constructed using the titration method in which water was added stepwise to the known proportions of the surfactant and the oil phase in the SMEDDS. Table 1 shows the composition of the SMEDDS for which we constructed a pseudo-three-component phase diagram using two different ratios of surfactant and oil phase (7/3 and 8/2, respectively).

#### 2.2.4. Loading of BMD in Microemulsion

The selected SMEDDS with incorporated BDP was prepared by first dissolving an excessive amount of BDP in the emulsifier (Kolliphor^®^ EL) and the oil phase (Capryol^®^ 90) in a ratio of 8/2. The prepared SMEDDS with BDP was stirred in a magnetic stirrer for one hour to ensure the complete dissolution of the drug. Later, water was added to the mixture using the titration method to obtain the microemulsion loaded with BDP. The microemulsion was thus prepared, containing 95% weight/weight (*w*/*w*) water, and poured into a centrifuge and centrifuged at 3500 rpm for 10 min. If a precipitate appeared after centrifugation, the amount of BDP in the original SMEDDS was reduced and the procedure described above was repeated. The optimal concentration of betamethasone dipropionate in the final microemulsion was 21.3 mg BDP/g SMEDDS.

### 2.3. Preparation of Hydrogel-Containing Microemulsion

For NCC-ALG or NCC-PEC hydrogels, BDP-loaded or unloaded SMEDDS diluted with water were added to one of two types of NCC, followed by alginate or pectin plus glycerol. The NCC/natural polymer ratio was 1/2, while SMEDDSS and glycerol content was 3 and 5%, respectively (Table 2). The drug content in BDP-loaded hydrogels was 0.64 mg/1 g of SMEDDS corresponding to the therapeutic dosage.

#### 2.3.1. DSC (Differential Scanning Calorimetry) Analysis

To determine the physical state of the BDP incorporated into hydrogels, we analyzed hydrogels with and without BDP using the DSC method (DSC1, Mettler Toledo, Columbus, OH, USA). Approximately 5 mg of sample was carefully transferred into a standard 40 µL Mettler Toledo aluminum pan, accurately weighed, covered with lid, and hermetically sealed. The closed pan was pierced just prior to the analysis to make a small pin hole. Samples were scanned in the temperature range from 25 °C to 250 °C at a heating rate of 10 °C/min and a nitrogen flow of 50 mL/min. The DSC curves were normalized to the sample mass. The melting points (Tm, peak) were determined using STARe V9.30 software (Mettler Toledo, Columbus, OH, USA).

#### 2.3.2. Rheological Study

The rheological properties of the different hydrogels were determined by a rotational method in a rheometer (Physica MCR 301, Anton Paar, Graz, Austria) with a conical disk diameter of 49.961 mm and a cone angle of about 2.001°. The applied mass of the sample was about 1 g, while the measurement temperature was 25 °C. The shear rate was increased from 1 to 100 (1/s), and the sample was initially left to rest for 30 s. The viscosity of the samples was determined using Physica software Rheoplus/32V3.62 based on the shear stress measurements.

Amplitude tests were used to determine the range of linear viscoelastic response in which the rheological properties of the substance are independent of the shear strain amplitude. The angular velocity was constant (10 rad/s), and the shear strain was continuously increased by 0.01–100%, measuring the elastic (G’) and plastic (G’’) moduli. Within this range, we then selected the parameters for further oscillation measurements during frequency tests.

Frequency tests were performed within the previously determined range of linear viscoelastic response. The changes in the elastic (G’) and plastic (G’’) moduli were monitored at an angular velocity of 100–0.1 rad/s at 0.1% deformation (determined during the amplitude tests).

#### 2.3.3. Stability Testing

The stability of the prepared hydrogels was investigated under room conditions (25 °C and 40% RH) and accelerated conditions (40 °C and 75% RH). An adequate number of BDP-loaded hydrogels was placed in a glass container and stored in two separate stability chambers for one month. After this storage period, the hydrogels were examined for the drug solid-state and viscosity as described in Section 3.2 and Section 3.3.

### 2.4. Film Preparation

Films were prepared from SMEDDS-hydrogels with and without embedded BDP using the drying method, which was optimized with a moisture analyzer (B-302, Büchi, Flawil, Switzerland). Approximately 3 g of each hydrogel sample was applied to a glass plate (8 cm × 8 cm) using a ZUA 2000 universal film applicator (Proceq, Schwerzenbach, Switzerland). The thickness of the applied hydrogel was 1 mm. The plate with the hydrogel was then placed in the moisture analyzer and dried at 32 °C. After a certain time, during which the mass of the dried sample did not further change (about 8 to 10 h), the films were obtained. The films were cut into uniform pieces and analyzed for residual moisture content and thickness.

#### 2.4.1. Film Residual Moisture Content Measurements

The tile with the film was weighed before and after drying. Drying was performed on 12 samples, in 3 parallels for each sample. Then, using the weighed values and Equations (1)–(3), the percentage of residual moisture in the films was calculated. In all cases, the percentage refers to a weight/weight (*w*/*w*)%.

(1) $$Remaining\;mass\;\left(\%\right)=\frac{m\left(film\right)}{m\left(hydrogel\right)}=\frac{m\;\left(tile+film\right)-m(tile)\;}{m\;\left(tile+hydrogel\right)-m\;(tile)}$$

(2) $$Dry\;matter\;\left(\%\right)=NCC\;\left(\%\right)+polymer\left(\%\right)+glycerol\left(\%\right)+SMEDDS(\%)$$

(3) $$Residual\;moister\;contnent\;\left(\%\right)=remaining\;mass\;\left(\%\right)-dry\;matter\;(\%)$$

#### 2.4.2. Film Thickness Measurements

The dried films were cut with a sharp blade to a size of 2 cm × 2 cm. The thickness of each film was measured in three separate places, using a digital caliper. Until further use, the films were stored in a plastic container with a lid, additionally wrapped with parafilm, at a temperature of 22 ± 2 °C.

#### 2.4.3. Measurements of the Water Retention Capacity of the Films

The ability to retain water evaporation in vitro was evaluated using the Tewameter^®^TM 300 (Courage&Khazaka GmbH, Cologne, Germany). Franz diffusion cells were filled with purified water and covered with cellulose acetate membranes (pore size 0.45 µm, Sartorius Stedim Biotech GmbH, Göttingen, Germany) previously soaked in purified water for 24 h, which provided physical support for the tested films. First, the basal water evaporation through the artificial membrane was measured. Then, the dried film (2 cm × 2 cm) was placed on the membrane and the water evaporation was again determined. After the film was moistened, the measured values began to increase. The measurement was stopped when the reading reached the basal value. The measurements were performed for films prepared from SMEDDS hydrogels with or without embedded BDP. The experiment was performed in triplicate. Data are expressed as averages.

#### 2.4.4. Morphological Evaluation of Films by Scanning Electron Microscopy

Scanning electron microscopy was used to determine the morphological differences of the prepared films. Films were placed on a carbon tape and examined with a Supra 35 VP, high-resolution scanning electron microscope (SEM) (Carl Zeiss, Oberkochen, Germany) at 1.0 kV acceleration voltage and different magnifications using a secondary detector.

### 2.5. Evaluation of Films and Hydrogels on Full-Thickness Pig Ear Skin

Films were additionally tested on full-thickness pig ear skin to evaluate their capability to decrease transepidermal water loss (TEWL) in vitro as a model skin membrane to upgrade the results obtained on artificial membranes. Furthermore, hydrogels as initially applied as a formulation on the skin were tested to determine their irritancy potential. Namely, using the nonperfused pig-ear test, the European Centre for the Validation of Alternative Methods procedure [25,26] classifies a tested formulation as a skin irritant when an absolute increase in TEWL ≥ 6 g/m^2^/h is observed [27].

Pig ears were obtained from a local abattoir and washed under tap water. The skin samples were prepared by removing the whole skin carefully from the underlying cartilage and inspected for visible skin lesions prior to use, with only intact, healthy-looking skin patches used. The skin was mounted on Franz diffusion cells with 9 mL 0.9% aqueous sodium chloride solution. The Franz cells (n = 4) were put into a water bath at 37 °C for 1 h, resulting in a skin surface temperature of 32 °C, then the basal TEWL (Tewameter^®^TM 300; Courage Khazaka, Cologne, Germany) was measured. Films were put onto the skin surface and the TEWL was measured after 10 and 30 min. As for hydrogels, after measuring the basal TEWL, approximately 40 mg of the formulation under test was put onto the skin surface for 1 h to air-dry. If some of the formulation remained as hydrogel, it was removed with a cotton swab. The changes in TEWL were measured after an additional 0.5 h.

The absolute change in TEWL was calculated as the difference between basal TEWL and that after the film/hydrogel exposure. The TEWL measurements on the pig ear skin were performed in a controlled environment, i.e., at the room temperature of 24.0 ± 1.0 °C and relative humidity of ~45%.

### 2.6. In Vitro Permeation Testing

In vitro permeation studies for BDP-loaded hydrogels were conducted using Franz diffusion cells (Verrerie Villeurbannaise, Villeurbanne, France) with a diffusion area of 0.785 cm^2^ and a receptor volume of 9 mL, with the exact volume considering for every single cell. Strat-M^®^ (Merck Milli-pore, Billerica, MA, USA) membranes were mounted between the donor and receptor compartments. Approximately 400 mg of sample was accurately weighted directly into the donor compartment. Phosphate buffer saline (PBS; pH = 7.4) containing 40% (*v*/*v*) ethanol was used as the receptor medium, constantly stirred at 400 rpm and thermostated at 32 ± 1 °C. Studies were performed under sink conditions. At predetermined time intervals (4, 6, 8, 10, 12, and 24 h), 600 μL aliquots were withdrawn from the receptor compartment and replaced by an equal volume of fresh, preheated receptor medium to keep the volume constant. Experiments were performed in four parallels. The BDP content in the receptor compartment was determined by HPLC analysis. The cumulative amount of BDP permeated through membrane (*Q_t_*) was plotted as a function of time and calculated according to Equation (4), where $c_{t}$ is the BDP concentration of receptor medium at each sampling time, *V_rm_* is the volume of receptor medium, *c_i_* is the BDP concentration at previous sampling times, *V_i_* is the sampling volume, and *M* represents the BDP mass at the donor compartment.

(4) $$Q_{t}=(c_{t}\times V_{\mathrm{rm}}+\sum_{i=0}^{t-1}c_{i}\times V_{i})/M$$

### 2.7. Cell Culture and Cell Proliferation Assay

Human keratinocyte cells (cell line NCTC 2544, ICLC, University of Genoa, Genoa, Italy) were cultured as adherent monolayers at 37 °C in a humidified atmosphere of 5% CO_2_. Cells were subcultured with trypsin/EDTA (Promega Corporation, Madison, WI, USA) when they reached 80–90% confluence. They were grown in Minimum Essential Medium complemented with 10% (*v*/*v*) fetal bovine serum (Gibco, Invitrogen, Waltham, MA, USA), 1% (*v*/*v*) non-essential amino acids, 1% penicillin/streptomycin mixture, 1% 2 mM L-glutamine, and 1% penicillin/streptomycin mixture. Cell culture reagents were from Sigma-Aldrich, St. Louis, MO, USA.

The effect of gNCC and pNCC dispersions on keratinocyte proliferation was evaluated using the MTS assay (Cell titer 96 Aqueous One Solution Cell Proliferation Assay; Promega, Madison, WI, USA). The assay is founded on the conversion of 3-(4,5-dimethylthiazol-2-yl)-5-(3-carboxymethoxyphenyl)-2-(4-sulfophenyl)-2H-tetrazolium, an inner salt, into the soluble formazan product by mitochondrial dehydrogenase enzymes in metabolically active cells. The assessment was performed according to the manufacturer’s procedure. Keratinocytes were seeded at a density of 0.5 × 10^−4^ cells per well in 96-well plates and left overnight to adhere. The cells were then treated with test samples, i.e., gNCC and pNCC diluted with cell medium. The final concentrations to which the cells were exposed were as follows: 0.5, 1.0, and 2.0 mg/mL. The cell proliferation was assessed 24 h after the addition of the test samples. The absorbance of formazan was read at 490 nm using a Safire2 microplate reader (Tecan Group AG, Männedorf, Switzerland). Experiments were performed in sextuplicate within three replicates. The cell proliferation was expressed as the absorbance ratio of treated cells to control cells, minus the absorbance of the test formulation in cell-free medium and medium alone, respectively.

### 2.8. Statistical Analysis

Statistical analysis was carried out using an independent samples Student’s *t*-test at the 0.05 level of probability in the case of film thickness after the drug incorporation, proliferation assay, and in vitro permeation test. Differences in outcomes of TEWL measurements on the artificial membrane and the pig ear skin were tested using mixed linear models due to the repeated measurements taken, including the fixed effects of the application mode. The analysis was performed using SPSS (IBM SPSS Statistics 25).

## 3. Results and Discussion

### 3.1. Hydrogel Preparation and Incorporation of BDP

Since BDP is practically insoluble in water, its incorporation in hydrogels presents a technological challenge. Therefore, BDP was first solubilized in a self-microemulsifying drug delivery system (SMEDDS) prior to its addition to hydrogels. SMEDDS are freely miscible with water, forming microemulsions, wherein the API (Active Pharmaceutical Ingredient) remains solubilized. The SMEDDS composition was chosen based on its optimal BDP solubilization capacity and its ability to form a microemulsion upon dilution with water media, which is present in hydrogels. Firstly, optimal lipids and surfactants exhibiting the maximum BDP solubility were chosen (Table 3).

Contrary to the previous reports [24,28], where oleic acid was determined as a promising oil media for BDP solubilization, the solubility of BDP in oleic acid was under the limit of detection. Instead, 55.8 mg/g of BDP was successfully dissolved in Capryol^®^ 90, which was chosen as an oil phase of the SMEDDS. The solubility of BDP in all surfactants was between 20.7 and 33.5 mg/g; therefore, all were individually mixed with the Capryol^®^ 90 and checked for microemulsion formation upon dilution with water. As hydrophilic cosolvent did not contribute to the BDP solubility in a meaningful way, its addition to the SMEDDS was omitted to lower the chance of BDP precipitation upon dilution of the SMEDDS with water.

**Figure 1:**
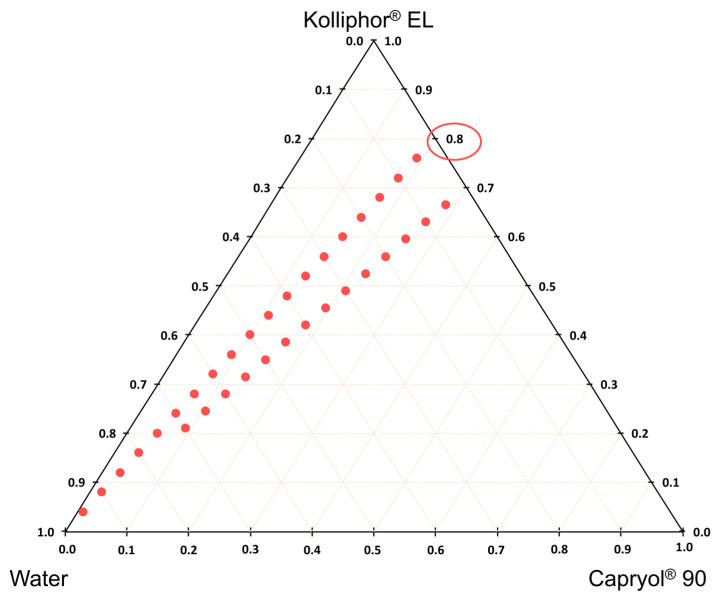
Three-component diagram for the chosen (8/2 and 7/3) ratios of surfactant (Kolliphor^®^ EL) and oil (Capryol^®^ 90) phase. Blue dots represent the presence of microemulsions upon dilution with purified water.

Among the tested mixtures, only Kolliphor^®^ EL/Capryol^®^ 90 formed microemulsions in contact with water (Figure 1). An 8/2 ratio of said excipients, loaded with 21.3 mg/g of BDP, was chosen as the final SMEDDS on the merit of its high BDP-saturated solubility and its ability to form microemulsions upon water dilution without BDP precipitation. A 3% addition of the SMEDDS loaded with BDP (as seen in Table 2) was therefore added to the hydrogels, which is equivalent to 0.064% (*w*/*w*) BDP, corresponding to the therapeutic ratio of 0.05% betamethasone (in a non-salt form) in the final film-forming gel composition.

### 3.2. DSC Analysis of Hydrogels

**Figure 2:**
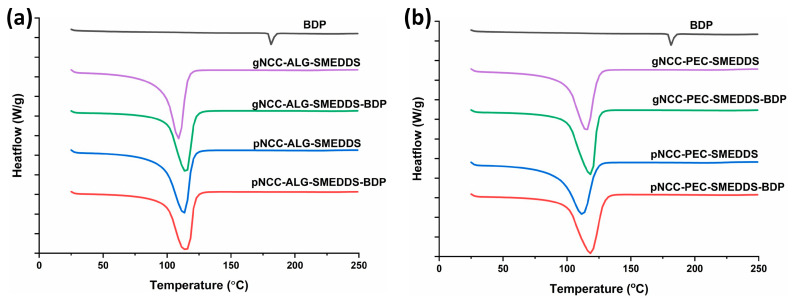
DSC heating curves of BDP-loaded and unloaded (**a**) NCC-ALG-SMEDDS and (**b**) NCC-PEC-SMEDDS hydrogels.

DSC analysis was performed for both types of hydrogels (alginate and pectin) (Figure 2). Namely, BDP exists in its natural state in crystalline form, and a sharp melting endotherm occurred at 178 °C in the DSC thermogram, which is in line with previously published results [29]. In addition, a broad endothermic peak can be seen in all hydrogels, ranging from 104–108 °C, representing water evaporation. No additional endothermic peak was observed in the hydrogels loaded with BDP, confirming that BDP remains dissolved upon incorporation into the hydrogels (Figure 2), which is extremely important for the transition of the drug into the skin and, consequently, for the achievement of the therapeutic effect.

**Figure 3:**
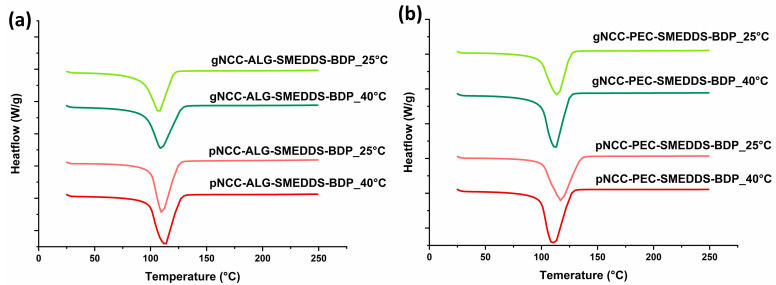
DSC heating curves of BDP-loaded (**a**) NCC-ALG-SMEDDS and (**b**) NCC-PEC-SMEDDS hydrogels after one month under room and accelerated conditions.

The BDP-loaded hydrogels stored for one month under room conditions and accelerated storage conditions showed a thermogram similar to the one obtained immediately after preparation (Figure 3). This indicates the physical stability of the BDP that remained dissolved in all types of hydrogels during the one-month storage period under both storage conditions.

### 3.3. Rheology of the Hydrogel Formulations

**Figure 4:**
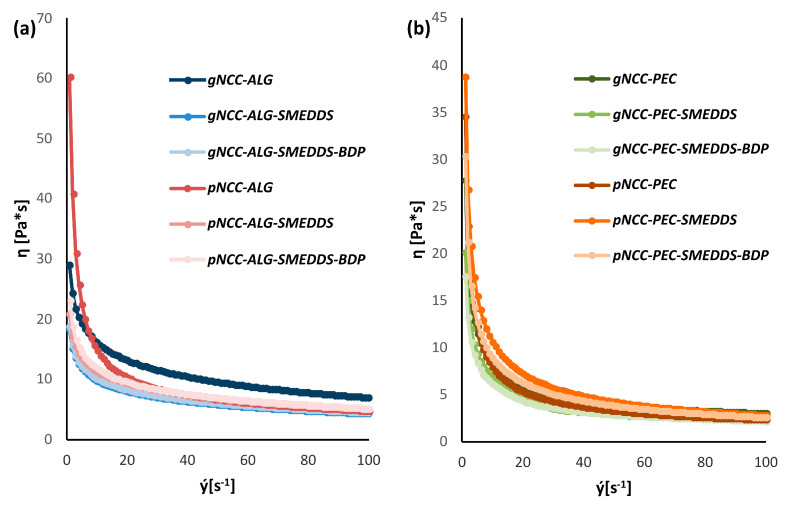
Rotational viscosity of hydrogel formulation for alginate (**a**) and pectin (**b**) samples.

Under macroscopic observation, all samples exhibited a typical appearance of hydrogels. Due to the properties of the input NCCs, the gNCC samples were pale gray in color, while the pNCC samples presented as whitish. All hydrogel samples were semi-opaque. As supported by rotational viscosity measurements, all prepared hydrogels could be classified as weak gels, exhibiting shear thinning behavior (Figure 4). Unsurprisingly, hydrogels containing pectin were less viscous, as its concentration was lower compared to that of alginate (3.5% vs. 5%).

**Figure 5:**
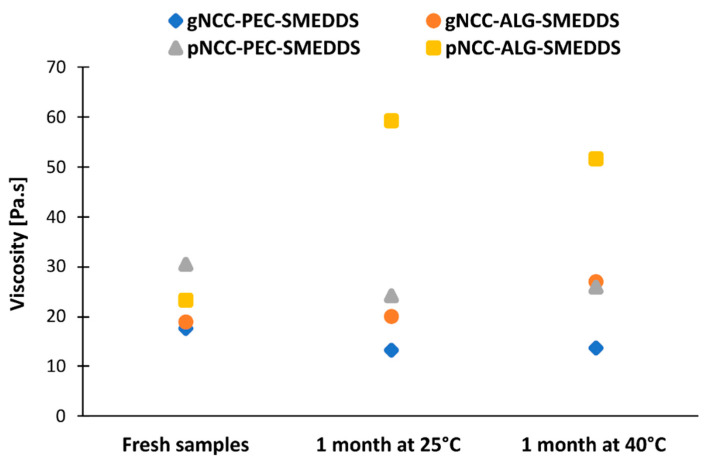
Rotational viscosity of hydrogel samples at shear rate 1 s^−1^ immediately after preparation vs. one month under room and accelerated conditions.

During the stability study, only minor changes were noted in the flow behavior of the hydrogels (Figure 5). The exception was pNCC-ALG-SMEDDS, which after one month in the stability chamber exhibited viscosity more akin to the fresh sample of pNCC-ALG than pNCC-ALG-SMEDDS. This could indicate a longer gelation time needed for polymers in the presence of the SMEDDS to achieve final rheological properties.

**Figure 6:**
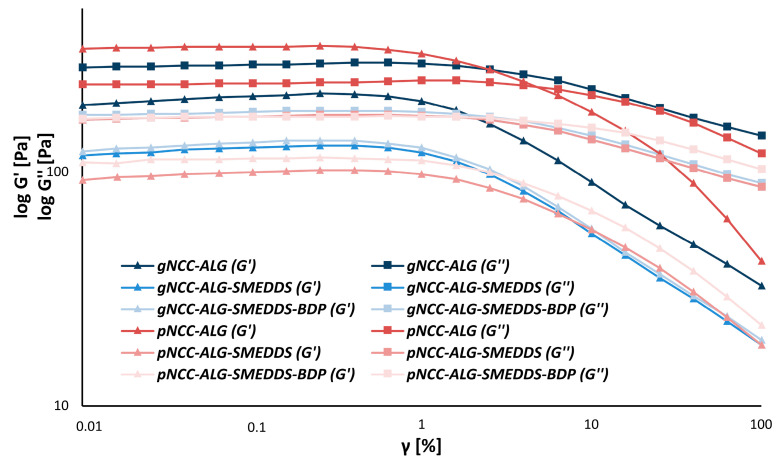
Amplitude sweeps of the nanocellulose-alginate hydrogels.

When observing the results of the amplitude sweeps in the LVE region (0.1%), however, some differences between the formulations could be noted. Namely, among alginate-based formulations, only pNCC-ALG exhibited gel-like behavior (G’ > G’’), while a liquid-like structure could be attributed to all other formulations (Figure 6).

**Figure 7:**
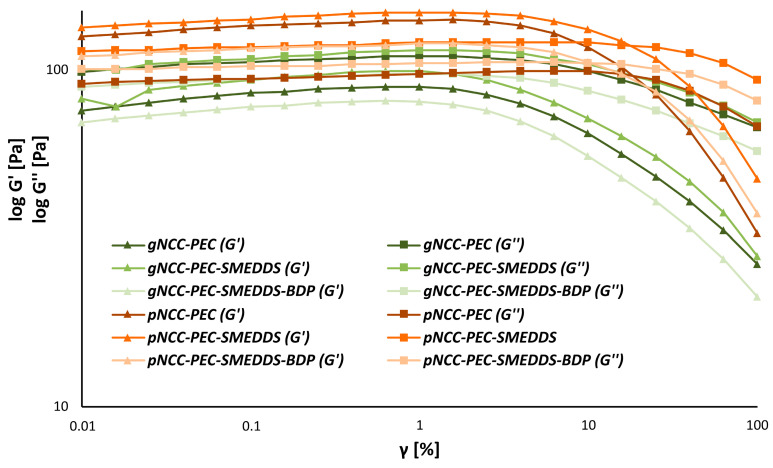
Amplitude sweeps of the nanocellulose-pectin hydrogels.

This was not the case for the pectin-based hydrogels (Figure 7). Here, all pNCC formulations exhibited gel-like behavior, regardless of whether they included the SMEDDS or the SMEDDS with BDP. On the contrary, gNCC formulations behaved more liquid-like. The exact structure of the NCC is therefore particularly important for gel formation. Unfortunately, to our knowledge, no studies on the gelation behavior of different nanocrystalline celluloses have so far been published.

**Figure 8:**
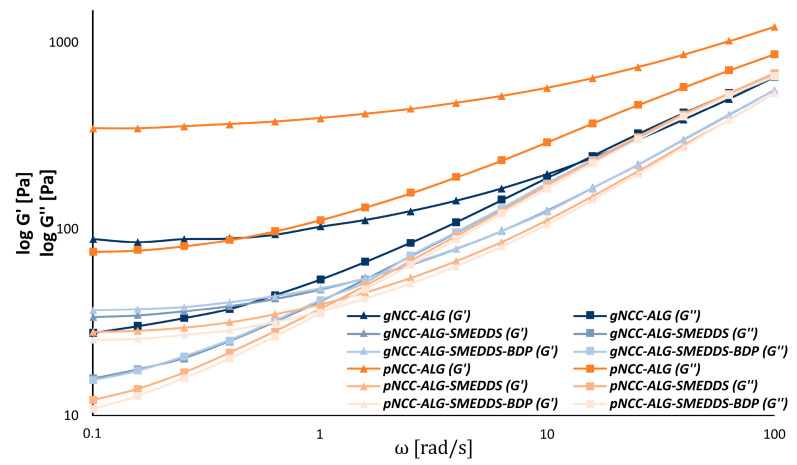
Frequency sweeps of the nanocellulose-alginate hydrogels.

The results from the frequency sweeps further confirmed the amplitude sweep results. While the gel structure of the pNCC-ALG hydrogel is deformed at larger ω values as gNCC-ALG, the addition of the SMEDDS (with or without BDP) to both formulations visibly disrupted the gel structure (Figure 8).

**Figure 9:**
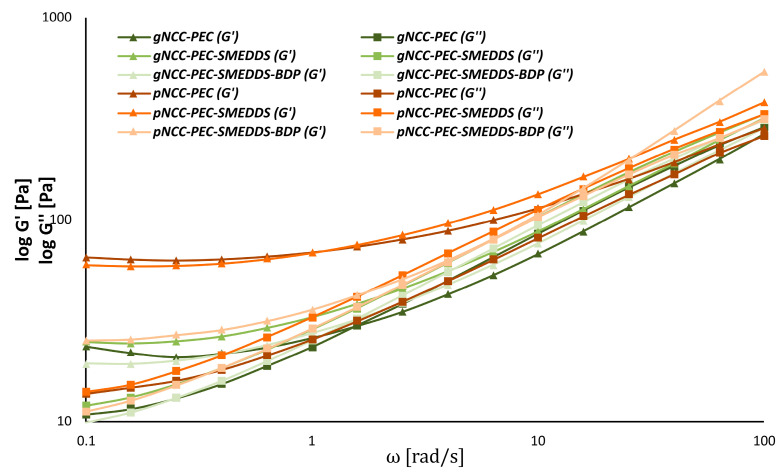
Frequency sweeps of the nanocellulose-pectin hydrogels.

For pectin-based formulations, pNCC consistently produced stronger gels over gNCC (Figure 9). This was true also for pNCC-PEC formulations with SMEDDS/SMEDDS-BDP, as no crossing of log G’ and G’’ could be observed within the range of measurements performed. Overall, the choice of the NCC is an especially crucial factor for hydrogel preparation, as it inherently influences the rheological properties of the final formulation.

### 3.4. Film Preparation and Evaluation

The main objective of the produced hydrogels is their transformation into films when applied to the skin. Therefore, the films of the hydrogels were prepared using the solvent evaporation method at 32 °C, mimicking the skin surface temperature. By applying hydrogels to glass plates and drying them in a dryer at 32 °C, we were able to demonstrate the film formation process in vitro. During the drying process, the water in the hydrogel evaporated, and the resulting film consisted of a three-dimensional polymer scaffold, serving as a reservoir for the subsequent drug release.

#### 3.4.1. Residual Moisture Content in the Films

**Figure 10:**
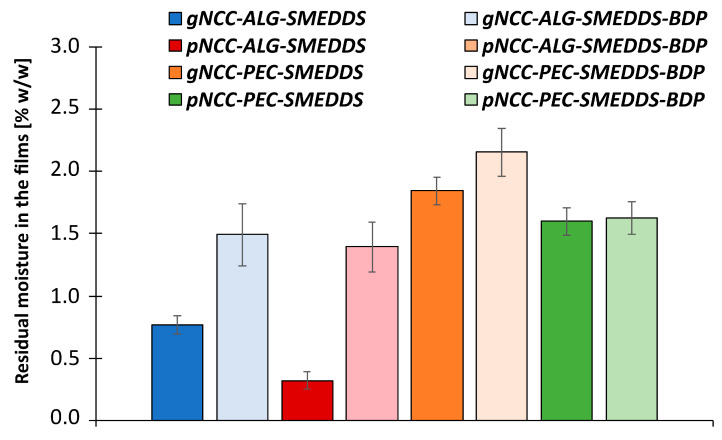
Residual moisture content (% *w*/*w*) in films or xerogels prepared from hydrogels. Results are expressed as mean ± standard deviation (n = 3).

After drying, the percentage of residual moisture in the films was measured. It varied from 0.32 to 2.15% (*w*/*w*) (Figure 10), depending on the weight of the film, showing that the residual water was entrapped in a three-dimensional network of polymer chains. In contrast to previous reports in which the moisture content in the pectin and alginate films was more comparable [30], our results show some difference between the two. Namely, the moisture content in the pectin films was significantly higher compared to the alginate films; this could be since the ratio of glycerol to polymers was higher in the pectine films than in the alginate films.

When comparing samples using different natural polymers, the percentage of residual moisture in films of the same composition also varied with respect to the NCC used (gNCC or pNCC). Here, we found that samples made from gNCC have a higher percentage of residual moisture compared to samples made from pNCC; individual differences between diverse types of NCC are therefore important to consider when formulating film-forming gels.

From the active-ingredient point of view, the samples with BDP contain a higher percentage of residual moisture than the samples of the same polymer composition without BDP, which is particularly evident in the alginate films. This shows that the active ingredient has a considerable effect on water binding within the gel.

It can be concluded that the percentage of residual moisture in the films is influenced by the choice of NCC and natural polymer, the interactions between the polymer chains, the percentage of polymer used (NCC and natural polymer), and the addition of the SMEDDS (without and with incorporated BDP).

#### 3.4.2. The Thickness of the Films

The incorporation of BDP as well as type of the polymer used affected the film thickness, which was greatest for the pNCC-ALG films loaded with BDP (0.078 ± 0.008 mm) and smallest for the pNCC-PEC films without BDP (0.046 ± 0.005 mm) (Table 4). It is evident that the pectin films are slightly thinner than the alginate ones, suggesting that the molecules can achieve a more compact arrangement when the pectin gel collapses. This could be due to the much lower estimated molecular weight of the pectin vs. the alginate used [31].

In addition, the incorporation of the drug into the films also noticeably affected their thickness, being greater than that of the films without BDP. Nevertheless, a significant difference was demonstrated only in the case of pectin-containing films.

#### 3.4.3. Water Retention Capacity of the Films

**Figure 11:**
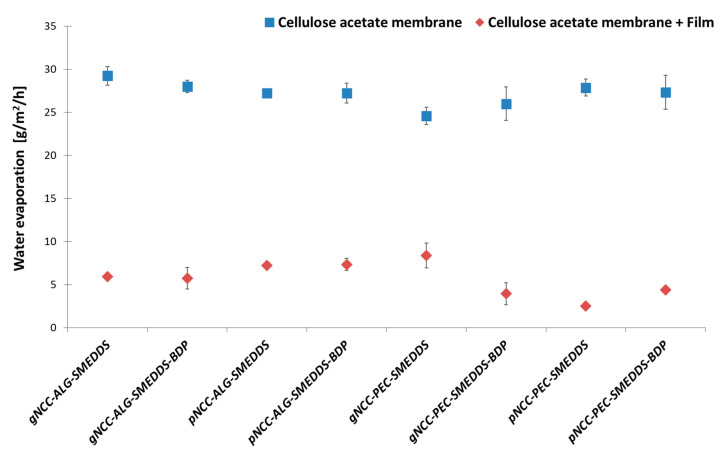
Water evaporation (g/m^2^/h) for cellulose acetate membrane (basal value) compared to cellulose acetate membrane with films (the water evaporation was measured prior wetting of films). Results are expressed as mean ± S.D. (n = 3).

Formulations that allow water retention in the skin are particularly beneficial in the treatment of AD. The prepared films reduced water evaporation in vitro (Figure 11) with a statistically significant difference (F (1, 24) = 4052.17, *p* < 0.001) compared to the cellulose acetate membrane (basal values), used as a carrier for films between the donor and acceptor compartments of Franz diffusion cells. Namely, the basal values ranged from 24.6 and 29.2 g/h/m^2^, while the measured values for the water uptake capacity of the films immediately before wetting ranged from 2.49 (pNCC-PEC-SMEDDS) to 8.38 g/m^2^/h (gNCC-PEC-SMEDDS). This shows that the ability of the membranes to decrease water improved significantly after the film was applied to the membrane. Furthermore, a difference in water retention capacity was observed across all tested films (F (11, 24) = 3.98, *p* = 0.002).

When comparing the influence of the polymer type used on the water retention and evaporation, we note some differences. In general, pectin samples in combination with pNCC show the highest ability to retain water, which is crucial for moisturizing the skin during AD therapy, with comparable results to alginate samples in combination with gNCC. This result can be related to the viscosity of these samples, being the highest and again comparable amongst gNCC-ALG and pNCC-PEC film-forming hydrogels. Namely, the higher viscosity of the hydrogels effectively reduces water evaporation from the skin and significantly increases the moisture content of the skin [24] and, consequently, also results in better film performance.

### 3.5. Evaluation of Transepidermal Water Loss after Film Application and Hydrogel Irritation Potential on Pig Ear Skin

Increased TEWL loss due to impaired skin barrier function is distinctive for atopic skin [32]. The idea behind hydrogels as film-forming systems upon dermal application on atopic skin is to provide a physical barrier that would improve skin barrier function and characteristics such as skin hydration due to less-pronounced TEWL. Consequently, dryness and sensitivity, the two main manifestations of atopic skin, would be recovered. To upgrade the results of the water retention capacity of the films, the direct effect of the films on TEWL was tested on pig ear skin.

**Figure 12:**
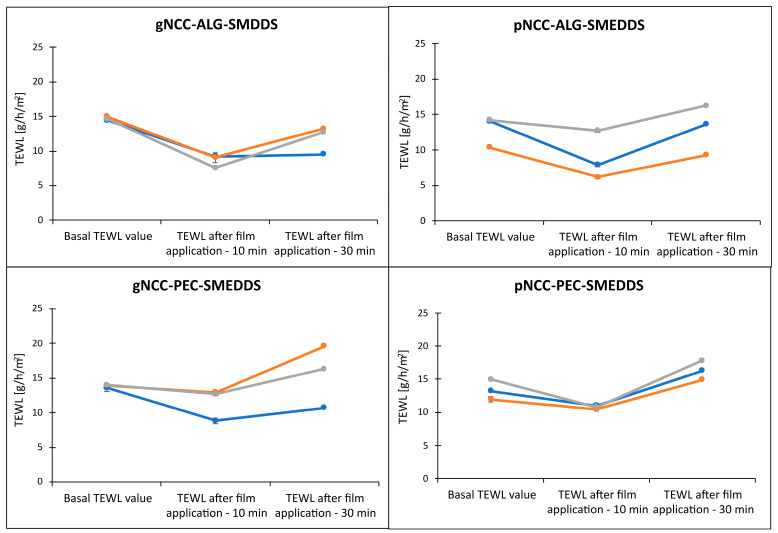
Changes of TEWL following film application for 10 min and 30 min. Three colored lines denote different parallel measurements on different patches of skin for each film formulation, as due to heterogenous nature of the skin, these cannot be averaged. Note: Individual TEWL value is an average of ten consequent and most stable TEWL values (indicated by S.D. ≤ 0.30) within one continuous 60 s long measurement.

As seen in Figure 12, following 10 min of film application, the TEWL was efficiently reduced regardless of the tested formulation, with the more pronounced decrease observed for alginate samples. Although the TEWL values started to increase after 30 min in the case of alginate samples, especially for gNCC-ALG, the results were still comparable to the basal values. Contrary to the results obtained on artificial membranes, the pectin samples less efficiently reduced TEWL, which in general had been even increased after 30 min for the basal values.

It can be postulated that in vitro evaluation on animal skin model membranes is crucial for a final appraisal of film effectiveness regarding their ability to decrease water loss (F (2, 7) = 40.63, *p* < 0.001). Although we observed some differences between the samples, suggesting improved performance of gNCC-ALG samples versus pNCC-PEC samples, the difference was not statistically significant (F (6, 16) = 1.34, *p* = 0.30).

**Figure 13:**
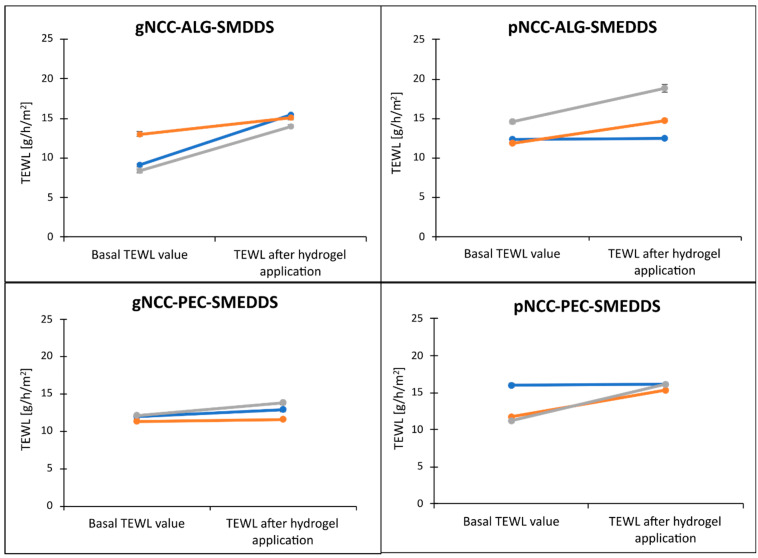
Increase in TEWL following exposure of the pig ear skin to the corresponding hydrogel for 1 h. Three colored lined denote three different parallel measurements on different patches of skin for each hydrogel formulation, as due to heterogenous nature of the skin, these cannot be averaged. Note: Measurements were performed 0.5 h after removal of hydrogel. Individual TEWL value is an average of ten consequent and most stable TEWL values (indicated by S.D. ≤ 0.30) within one continuous 60 s long measurement.

The aspect of the biological acceptability of the formulation developed is crucial for actual in vivo application, as the therapeutic effects must be accomplished without any adverse skin effects. The irritancy potential of hydrogels was therefore additionally addressed, especially due to the SMEDDS being incorporated since surfactants could interact with stratum corneum lipids and lead to disrupt barrier function. The pig-ear test closely resembled the protocol for the assessment of the films’ capacity to reduce TEWL. Moreover, the hydrogels were additionally evaluated, as they represent the initial formulation applied on the skin. The results of the in vitro nonperfused pig-ear test for determination of the acute skin irritation of the hydrogel formulations are shown in Figure 13.

The basal TEWL values of the skin samples (prior to hydrogel application) were compared to TEWL values following exposure of the pig skin to test hydrogels for 1 h. These increases in TEWL ranged from 2.1 g/h/m^2^ to 5.6 g/h/m^2^ for alginate-based formulations and 0.19 g/h/m^2^ to 4.89 g/h/m^2^ for pectin samples. The highest TEWL increase, just slightly below 6 g/h/m^2^, was observed for two tested gNCC-ALG samples, i.e., 5.6 and 5.3 g/h/m^2^. Nevertheless, the hydrogel formulations provoked a minor absolute increase in TEWL, enabling us to classify those hydrogels as nonirritant (F (1, 8) = 21.77, *p* = 0.002) [27]. However, we did not observe statistically significant differences regarding the type of hydrogel applied (F (3, 8) = 1.74, *p* = 0.24). In addition, apart from the NCCs as innovative ingredients for which cytotoxicity assessment was performed (Section 3.7), toxicological information was collected (Table 5) for other ingredients from the safety data sheets provided by the manufacturers. All excipients are widely used in pharmaceutical formulations and are generally recognized as safe with low acute toxicity reported in animals. Importantly, all ingredients utilized in formulations here were designated as nonirritant or mild irritants at most.

### 3.6. In Vitro Permeation Test

**Figure 14:**
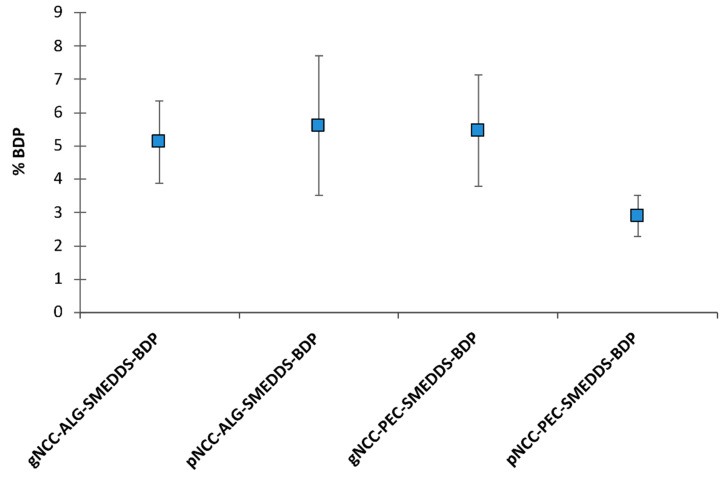
The amount of BDP permeated after 24 h. Results are expressed as mean ± S.D. (n = 4).

Within the development phase of dermal delivery systems, in vitro permeation testing is essential for the prediction of dermal absorption in vivo; however, quite a few limitations are faced when operating with human or animal skin. As an alternative, the Strat-M^®^ membrane can be used as an artificial membrane. It is composed of multiple layers mimicking skin structure with, in brief, a tight top layer resembling the stratum corneum as the main skin barrier for dermal absorption, two layers resembling dermis, and a final more open and diffusive layer [33,34]. Thus, in vitro permeation from BDP-loaded hydrogels (0.64 mg/g) was studied using Strat-M^®^ membranes. Regardless of the BDP-loaded hydrogels tested, no standard permeation profiles were observed; thus, the amounts of BDP permeated (%) after 24 h are presented in Figure 14.

**Figure 15:**
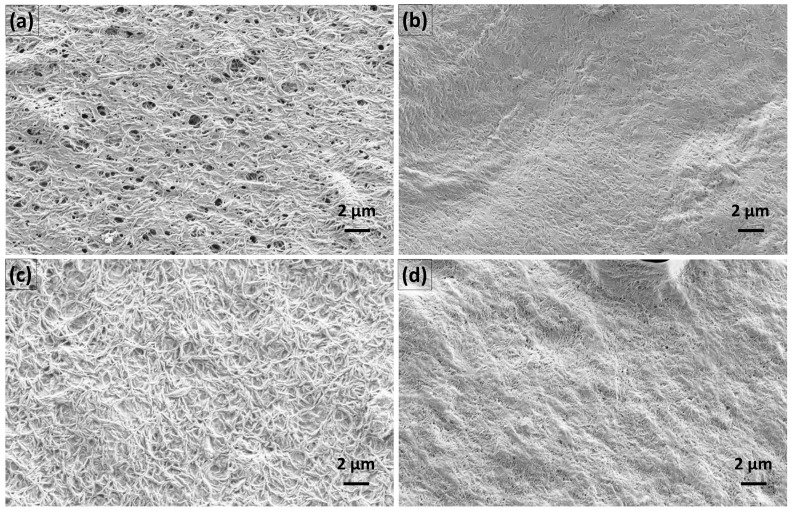
SEM images of (**a**) gNCC-ALG-SMEDDS, (**b**) pNCC-ALG-SMEDDS, (**c**) gNCC-PEC-SMEDDS, and (**d**) pNCC-PEC-SMEDDS films at 10,000× magnification for all samples.

Namely, in the case of hydrogels with alginate, the BDP was firstly observed in the receptor medium following 12 h or 8 h contact with the membrane, with 2.53 ± 1.37% and 1.87 ± 0.06% of the BDP permeated in the case of the gNCC and pNCC hydrogels, respectively. After 24 h, 5.11 ± 1.23% permeated from the gNCC-ALG hydrogel, whereas a slightly higher amount was permeated in the case of the pNCC-ALG hydrogels, i.e., 5.61 ± 2.09%. As for hydrogels with pectin, the BDP was detected after 6 h in the case of the gNCC-PEC hydrogels (1.20 ± 0.57), finally resulting in 5.46 ± 1.68% of the BDP in the receptor medium after 24 h, whereas in case of the pNCC-PEC hydrogels, the BDP was not released before 12 h (1.37 ± 0.39%), and at the end of experiment 2.90 ± 0.61% of the BDP permeated in the receptor medium. The amount of BDP permeated from the pNCC-PEC hydrogel was not statistically different compared to the pNCC-ALG hydrogel, whereas a statistically significant difference was observed when comparing it to the gNCC-hydrogel either with alginate or pectin (*p* < 0.05). The results, even though not straightforward, can be explained by the differences in NCC morphology. Namely, gNCC, with thicker and longer fibers (up to 15 × 300 nm, as per the manufacturer’s specification), forms a 3D lattice with bigger pores, whilst the pNCC (up to 7.5 × 150 nm fibers) network forms smaller pores, as also confirmed by SEM images (Figure 15). Namely, distinct variations in the morphology of the prepared films, primarily associated with the specific type of NCC used, can be observed. As depicted in Figure 15, the gNCC-based samples exhibit considerably larger pores, especially those in combination with alginate, whereas for pNCC-based films, a smooth surface is observed. However, what is the most outstanding is the prolonged release observed for all systems tested, indicating that irrespective of type of NCC or polymer used, a scaffold is formed that enables the prolonged release of the incorporated BDP, acting as a reservoir for the drug that could contribute to less frequent administration, leading to better patient compliance.

### 3.7. Cell Proliferation Assessment

The cell proliferation was evaluated by MTS assay for both types of NCC as relatively novel and, above all, the main excipients in nanocellulose-based film-forming hydrogels.

**Figure 16:**
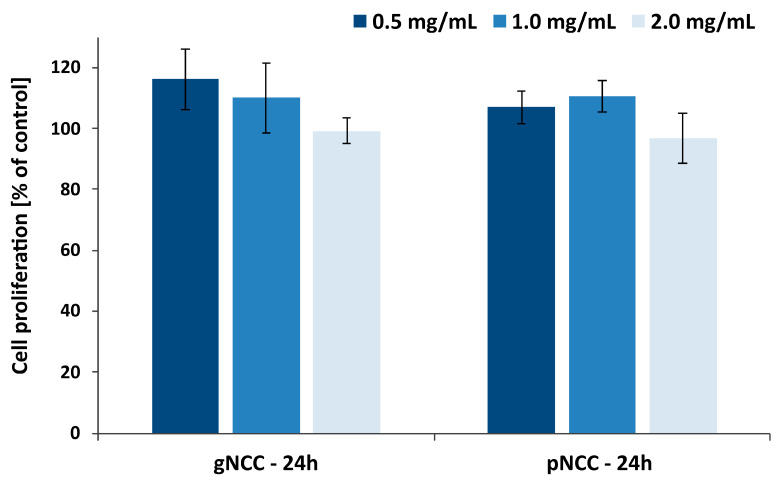
Keratinocyte proliferation after 24 h treatment with gNCC or pNCC dispersion. Data are expressed as mean ± S.D. with the results presented being relative to the proliferation of untreated cells.

In general, NCC is derived from different cellulose sources by means of various processing methodologies [35], and at this point, it is not an excipient generally recognized as safe. Therefore, cytotoxicity assessment is crucial for specific types of NCC; e.g., it was reported that NCC from the production waste of rubber-wood and kenaf-bast fibers did not exhibit cellular toxicity upon exposure to different cell lines [36]. For both types of NCCs used within our study, the cell proliferation was assessed by MTS metabolism following long-term (24 h) exposure in final concentrations of 0.5, 1, and 2 mg/mL. The results (Figure 16) show a clear profile with no considerable impact on cell proliferation, being even above 100% for both NCC types with only a subtle and statistically insignificant decrease observed for the highest concentration tested. The high proliferation values at the lower concentrations tested could be to some extent related to the observed precipitates of NCCs when dissolved in cell medium at 37 °C, while the effect on cell proliferation prevailed at the highest concentration tested. The obtained results confirm that both types of NCC used within this study pose no physical threat to skin cells when used as excipient for film-forming hydrogels for dermal application.

## 4. Conclusions

Two different types of NCC from cellulosic sources were proven suitable for the development of film-forming hydrogels with alginate or pectin, also in the view of biological acceptability. BDP was successfully incorporated into NCC-based hydrogels at therapeutic dosages and remained dissolved using a SMEDDS. Using the solvent evaporation method, flexible films were formed at 32 °C and significantly reduced in vitro water evaporation through an artificial membrane as well as through pig ear skin. In addition, a prolonged release of BDP was observed, indicating that the film-forming hydrogels act as a reservoir for the drug, allowing the benefit of less frequent administration. NCC-based film-forming hydrogels therefore present novel prospective delivery systems for AD treatment with the added benefit of sustainability. Further research work should focus on elucidating the most important properties of NCC materials for film-forming hydrogels.

## Figures and Tables

**Table 1:** Composition of SMEDDS for which the pseudo-three-component phase diagram was created.

Oil Phase	Surfactant Phase	Surfactant/Oil Phase Ratio
Capryol^®^90	Tween^®^ 20	7/3 and 8/2
	Tween^®^ 80	
	Labrasol^®^	
	Polyethylene glycol 400	
	Kolliphor^®^ EL	

**Table 2:** Composition of hydrogels from NCC and alginate/pectin in % *w*/*w*, where the red color represents the excipients added to the alginate hydrogels only and the blue color represents the composition of the pectin hydrogels. All other excipients indicated in black were added to both types of hydrogels.

	gNCC		ALG	PEC	SMEDDS	SMEDDS-BDP	Glyc.
	ALG or PEC						
gNCC-ALG/PEC	2.5%	1.75%	5%	3.5%	/	/	5%
gNCC-ALG/PEC-SMEDDS	2.5%	1.75%	5%	3.5%	3%	/	5%
gNCC-ALG/PEC-SMEDDS-BDP	2.5%	1.75%	5%	3.5%	/	3%	5%
	pNCC						
pNCC-ALG/PEC	2.5%	1.75%	5%	3.5%	/	/	5%
pNCC-ALG/PEC-SMEDDS	2.5%	1.75%	5%	3.5%	3%	/	5%
gNCC-ALG/PEC-SMEDDS-BDP	2.5%	1.75%	5%	3.5%	/	3%	5%

The addition of purified water was adjusted according to the water content (*w*/*w* %) already present in the gNCC.

**Table 3:** Solubility of BDP in oils and surfactants.

Scheme		Saturated Solubility of BDP (mg/g)
Oil phase	Capmul MCM C8	24.8
	Capryol 90	55.8
	Isopropyl myristate	*
	Oleic acid	*
	Plurol oleique	4.3
Surfactant phase	Tween 20	24.9
	Tween 80	22.9
	Labrasol	33.5
	Kolliphor EL	20.7
Hydrophilic cosolvent	Polyethylene glycol 400	23.4

* Under LOD.

**Table 4:** Thickness of films prepared from hydrogels. Results are expressed as mean ± standard deviation (n = 5).

Sample Name	Film Thickness (mm)
gNCC-ALG-SMEDDS	0.058 ± 0.004
gNCC-ALG-SMEDDS-BDP	0.064 ± 0.015
pNCC-ALG-SMEDDS	0.062 ± 0.008
pNCC-ALG-SMEDDS-BDP	0.078 ± 0.008
gNCC-PEC-SMEDDS	0.052 ± 0.004
gNCC-PEC-SMEDDS-BDP	0.068 ± 0.008
pNCC-PEC-SMEDDS	0.048 ± 0.005
pNCC-PEC-SMEDDS-BDP	0.060 ± 0.007

**Table 5:** Toxicity and irritancy of the hydrogel ingredients obtained from the safety data sheets of the chemicals used, as provided by the manufacturers.

Ingredient	Toxicity		Irritancy
	LD_50_ Oral	LD_50_ Dermal	
Alginate	>5 g/kg (rat)	/	No skin irritation
Pectin	>5 g/kg (rat)	/	Prolonged contact with dry powder may cause mild skin irritation.
Glycerol	12.6 g/kg (rat)	>10 g/kg (rabbit)	Mild skin irritation (rabbit)
Capryol 90	>5 g/kg (rat)	>5 g/kg (rat)	Mild skin irritation (rabbit)
Kolliphor EL	>6.4 g/kg (rat)	>5 g/kg (rat)	No skin irritation (rabbit)

LD_50_, median lethal dose; /, no data available.

## Data Availability

The data presented in this study are available upon request from the corresponding author.
